# The ghosts of HeLa: How cell line misidentification contaminates the scientific literature

**DOI:** 10.1371/journal.pone.0186281

**Published:** 2017-10-12

**Authors:** Serge P. J. M. Horbach, Willem Halffman

**Affiliations:** Radboud University, Institute for Science in Society, Nijmegen, The Netherlands; KU Leuven, BELGIUM

## Abstract

While problems with cell line misidentification have been known for decades, an unknown number of published papers remains in circulation reporting on the wrong cells without warning or correction. Here we attempt to make a conservative estimate of this ‘contaminated’ literature. We found 32,755 articles reporting on research with misidentified cells, in turn cited by an estimated half a million other papers. The contamination of the literature is not decreasing over time and is anything but restricted to countries in the periphery of global science. The decades-old and often contentious attempts to stop misidentification of cell lines have proven to be insufficient. The contamination of the literature calls for a fair and reasonable notification system, warning users and readers to interpret these papers with appropriate care.

## Introduction

The misidentification of cell lines is a stubborn problem in the biomedical sciences, contributing to the growing concerns about errors, false conclusions and irreproducible experiments [1, 2]. As a result of mislabelled samples, cross-contaminations, or inadequate protocols, some research papers report results for lung cancer cells that turn out to be liver carcinoma, or human cell lines that turn out to be rat [3, 4]. In some cases, these errors may only marginally affect results; in others they render results meaningless [4].

The problems with cell line misidentification [5] have been known for decades, commencing with the controversies around HeLa cells in the 1960s [6–10]. In spite of several alarm calls and initiatives to remedy the problem, misidentification continues to haunt biomedical research, with new announcements of large-scale cross-contaminations and widespread use of misidentified cell lines appearing even recently [11–13]. Although no exact numbers are known, the extent of cell line misidentification is estimated between one fifth and one third of all cell lines [4, 14]. (Although currently only 488 or 0.6% of over 80,000 known cell lines have been reported as misidentified, most cell lines are used infrequently [15].) In addition, misidentified cell lines keep being used under their false identities long after they have been unmasked [16], while other researchers continue to build on their results. Considering the biomedical nature of research conducted on these cell lines, consequences of false findings are potentially severe and costly [17], with grants, patents and even drug trials based on misidentified cells [18]. Several case studies performed by the International Cell Line Authentication Committee (ICLAC) highlight some of the potential consequences of using misidentified cell lines [19, 20]. Especially in the last decade, the gravity of the problem has been widely acknowledged, with several calls for immediate action in journal articles [3, 12, 21–23], requirements for grant applications (e.g. [24, 25]) and even an open letter to the US secretary of health [26].

The current calls for action and remediation activities are almost exclusively concerned with avoiding future contaminations, such as through systems for easier verification of cell line identities. Various solutions have been proposed [27–29], among others employing genotypic identification through short tandem repeats (STR) [30]. In addition, authors are expected to check overviews of misidentified cells (such as [12, 15, 27, 31]) before conducting their experiments. However, little attention is currently paid to the damage that has already been done through the past distribution of research articles based on misidentified cells. Although systems such as retractions and corrections are available to alert other researchers of potential problems in publications, these systems are rarely used to flag problems with cell lines [20, 32]. Even if future misidentifications could be avoided completely–which is not likely given the track record of earlier attempts–these ‘contaminated’ articles will therefore continue to affect research.

Before any action can be taken, it is essential that we get a sense of the size and nature of the problem of contaminated literature. This raises several questions. First, how many research articles have been based on misidentified or contaminated cell lines? How wide is their influence on the scientific literature? Second, what can we say about origins and trends in the contaminated literature? Is the problem getting better, or restricted to peripheral regions of the world’s research, where perhaps protocols are less strict? Third, what could be appropriate ways to deal with the contaminated literature? To answer these questions, we searched the literature for research papers using cell lines that are known to have been misidentified. In order to put the results of this search in perspective, we analysed the precise complications of misidentification for three particular cell lines.

### The process of distributing cell lines

**Fig 1:**
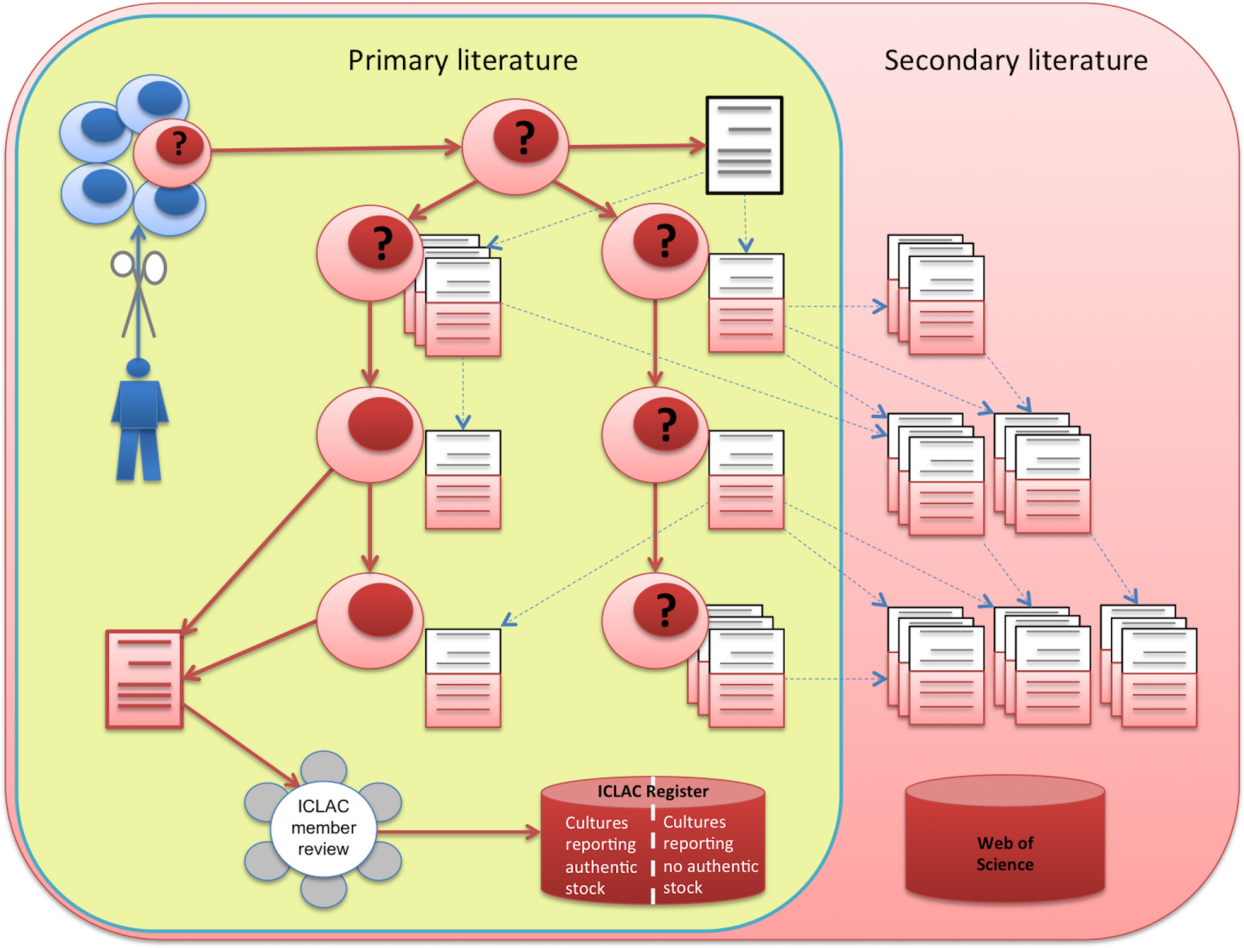
The creation, distribution and literature of a cell line: A cultured sample of cells (blue cells) may produce an immortal cell line (red cells), sometimes announced in ‘an establishing paper’ (in white). Cells may then be distributed to other researchers and reported in research papers, the ‘primary literature’. If misidentification of cells is reported in ‘a notifying paper’ (in red, bottom left), this may raise questions about the entire cell line (question marks) and the papers based on it, since misidentification commonly occurs at the source. Notifying papers should be reported to ICLAC, which will decide whether cell lines should be added to the ICLAC misidentified cell line register. Meanwhile, the contaminated primary literature is cited (dotted lines) by ‘secondary literature’, spreading the contamination further.

To study the scale of literature contamination, we need to understand the process of setting up, distributing, and publishing about cell lines. This process is illustrated in Fig 1.

Briefly, setting up a novel cell line commences with a tissue sample from an organism, human or other. If this culture grows successfully, the establishment of a new cell line is sometimes reported in what we will call an ‘establishing paper’. Subsequently, scientists may share or obtain this cell line, either via their personal network or via cell banks. These scientists may perform research on this cell line and publish their findings in scientific journals, thereby setting up what we call the *primary literature* based on a cell line.

At some point, a cell line may be found to be misidentified. This observation may be published in a ‘notifying paper’, leading to a registration in the International Cell Line Authentication Committee’s (ICLAC) database of cross-contaminated or misidentified cell lines [12]. Based on available data, cell lines can be added to one of two tables. The first is reserved for cell lines with no known authentic stock, and the second for cell lines where authentic stock is known to exist.

In this paper, we focus on the first category only: cell lines without any reported original stock. In this case, it must be assumed that all primary literature could be based on false grounds and should at least be treated with caution. In addition, we tried to estimate the size of the *secondary literature*: those articles referring to the primary literature, hence potentially building on questionable materials.

## Materials and methods

### Gathering data

Version 8.0 of the ICLAC list of misidentified cell lines [12] was published in December 2016 (http://iclac.org/databases/cross-contaminations/). From this list we only used table 1, listing those cell lines for which no authentic stock of the originally presumed cell line is reported. This list holds 451 cell lines. The identification of research articles using any of these misidentified cell lines was more difficult than expected. Although any article based on research using one of the listed cell lines supposedly mentions this cell line, the information is frequently not incorporated in one of the searchable fields in large databases (such as title, abstract or keywords), not even in otherwise well-documented biomedical databases such as PubMed. Therefore, the exact number of papers based on misidentified cell lines cannot be established. However, we could identify articles that either refer to the establishing article of a misidentified cell line, or that name the cell line in their title, abstract or key words. This search was carried out within the Web of Science database, because this platform allows for detailed citation analysis. We used the following two search methods to obtain conservative estimates of the number of research articles based on misidentified cell lines:

#### Method 1

For any cell line on the ICLAC list we tried to find an original article that reports the establishment of the cell line. These ‘establishing articles’ were searched using the Cellosaurus database [15], and subsequent references herein to the German collection of microorganisms and cell lines (DSMZ) database, the American Type Culture Collection (ATCC) database, and the European Collection of Authenticated Cell Cultures (ECACC) database. The DSMZ, ATCC and ECACC databases were consulted to check for references to any establishing article on one of the cell lines. Establishing articles were found for 255 cell lines. Subsequently, the original articles found in this fashion were searched in the Web of Science database. All references to the establishing articles were collected. We considered a reference to this original article as a good proxy for the usage of a cell line, since typically the original papers are focused on reporting the establishment of the cell line only (as checked in our three case studies described below).

It could be argued that we went too far back in time. It is a common phenomenon that articles have a limited ‘lifespan’, the time during which they receive citations, which would mean that older establishing articles would currently be poorly cited as the cell lines they announced are no longer relevant. To test for this we considered the citation lifespan of all establishing articles published before 1980. They turned out to have an average citation lifespan of over 40 years and the majority of them still received citations in 2016 or 2017. Hence we believe there are good reasons to be inclusive of even relatively old establishing papers.

#### Method 2

We searched the WoS database for all articles stating the names of one of the 451 listed cell lines in their title, abstract or keywords, as well as one of the words: ‘cell(s)’ or ‘cell line(s)’. In order to provide for more accurate search results, we only searched articles in the 25 research fields (as defined by WoS) that were most common among the results of the first search method. Thereby we excluded articles that use misidentified cell lines, but were classified in fields in which research on cell lines is less common. We note that even though the research areas as defined by WoS may overlap, classifying articles in more than a single research area, this has not lead to double counting in our analysis: all articles included in one of the 25 identified research areas were only counted once.

### Verification of data

Several strategies were employed to verify the validity of the data and to reduce the number of ‘false positives’ (i.e. those articles ending up in our sample, without reporting on research with misidentified cell lines). Regarding search method 1, we closely verified all establishing articles that resulted in at least 100 hits of primary articles in our database (n = 41). In this verification we found one article that was actually a notifying paper instead of an establishing paper and hence deleted it from our search. In addition, four articles were found that report on the establishment of several cell lines, some of which are not listed in the ICLAC database. In two of these cases, the establishing article reports on both the establishment of the contaminated cell line as well as the contaminating cell line (in the cases of EJ138 and HPB-MLT). We decided to not delete those establishing articles from our database, because they yield only few false positives (order of magnitude of tens) in our database.

Regarding search method 2: Due to some names of cell lines that could easily be confused with other meanings (such as ‘WISH’, ‘CaVe’ or ‘EU-1’), this search created noise. Therefore we used an iterative process to delete this noise. This process proceeded as follows: we observed a random sample of 100 articles, sampled by ordering the articles on publication data and selecting every 200^th^ hit. We checked whether the articles found actually used one of the listed cell lines, this was done by first reading the abstract of the found articles and, in case of doubt, further consulting the full text. If the research reported in the article did not use one of the listed cell lines, the search term that found this article was either replaced by: “*name of cell line* cell*” (e.g. “WISH cell*” instead of “WISH”), which was done in 26 cases, or the search term was deleted from the query (in cases of very generic words such as in the case of the ‘OF’ cell line), which was done in 15 cases (see Supporting Information S1 File for a list). (In this search, the asterisk (*) signifies a wildcard, i.e. the term ‘cell*’ will find any word starting with ‘cell’.) We continued this process until the samples did not contain false positives due to structural issues in the search terms.

Subsequently, the process of randomly selecting 100 articles was iterated four times and was executed independently by both authors. Concluding from the results in the random samples, our search method provides reliable results. Nevertheless, the results inevitably contain remaining false positives, the extent of which is estimated to a maximum of 10% of the contaminated primary literature, judged by our verification through random samples of the set, in which 6.5% of the articles was found to consist of false positives The set of false positives contains, among others, articles using cells in the ICLAC register that are nonetheless reported with their correct origin (as reported for KB cells by Vaughan et al. [20]).

All claims in this article are based on a dataset of articles found through either method 1 or 2. Both searches were performed without additional software tools, but with manual searches working with complex Boolean search strings. To gather information on the secondary literature, i.e. those articles citing the articles in the primary literature, we used the standard WoS ‘citation report’. In the secondary literature we excluded self-citations in order to observe the actual ‘spreading’ of the contaminated literature. The exclusion of self-citations is a standard option in the WoS’ citation report.

### Case studies

In order to verify the collected data and to get a deeper understanding of how knowledge based on misidentified cell lines spreads through the literature, we performed three case studies in which we tracked the publications concerning a single cell line or a family of cell lines. All three are misidentified cell lines for which no original stock was reported and were selected at random from the ICLAC database. The case studies were performed on the cell lines: ALVA-31, a family of thymic cell lines (F2-4E5, F2-5B6, P1-1A3 and P1-4D6), and JCA-1. The results of the case studies indicate that our search method indeed renders accurate data, with only very few ‘false positives’, and rather conservative estimates.

### Analyses of the contaminated literature’s origins

We performed several analyses on the contaminated primary literature’s origins based on WoS data, analysing their temporal and geographical origin and the distribution over research areas. The development over time uses the WoS publication date of the definitive version of the article; hence electronic versions may have been published prior to this date. The WoS goes back to 1945, but is incomplete for the first decades of the database. For the geographical origin of the research records, we employed the WoS category ‘Country/Territory’, which is based on the affiliation of the authors. The origins of the contaminated primary literature are compared with the total literature on research involving cell lines. This total literature comprises the articles that mention any word starting with ‘cell’ (i.e. cell, cells, cellular, etc.) in their title, keywords or abstract (hence not only misidentified cells), and belong to the 25 WoS-defined research areas that were most common among the dataset of contaminated primary literature. This reference group was also used to estimate what fraction of the relevant total literature is contaminated (see under the heading Contamination of the scientific literature).

## Results

### Contamination of the scientific literature

Using ICLAC’s *Database of Cross-Contaminated or Misidentified Cell lines* [12], we searched the scientific literature with the *Web of Science* (WoS) [33] to identify research articles based on misidentified cell lines. Using complementary search strategies (see methods), we were able to identify 32,755 articles (on August 4^th^, 2017) based on cell lines that are currently known to be different from the cell lines reported in these publications. As we only searched for cell lines known to be misidentified, this constitutes a conservative estimate of the scale of contamination in the primary literature. Moreover, to avoid false positives, we excluded several cell lines, such as the ones with non-unique identifiers or the cell lines for which verified stock is still in circulation. With non-unique identifiers we refer to names of cell lines that do not only refer to the cell line but (potentially) also to other phenomena. For example the case of the ‘OF’ cell line or the ‘WISH’ cell line. With ‘non-unique identifier’ we hence do not refer to cell lines that have multiple names or names with multiple spellings (such as the Intestine 407 cell line, which is also called ‘Intestine407’, ‘Int-407’ and ‘Int407’). In cases of multiple spellings of cell line names, we stuck to the spelling indicated in the ICLAC database. Thereby we probably missed many articles using these cell lines in search method 2, again leading to conservative estimates.

In addition, research based on misidentified cell lines has a wide impact on the scientific literature, as it appears that these research papers are comparatively highly cited. WoS does not allow for precise total numbers, but we can give indications of this ‘secondary contamination’ of the literature. Analysing citations to primary contaminated articles, we found 46 papers with more than a thousand citations and over 2600 contaminated articles with over a hundred citations. Furthermore, over 92% of the contaminated papers are cited at least once, which is more than average for biomedical literature [34]. In total, we can conservatively estimate the citations to the primary contaminated primary literature at over 500,000, excluding self-citations, thereby leaving traces in a substantial share of the biomedical literature. Even though it is clear that articles may receive citations for many reasons, including negative or even ritual citations, and hence not all citing articles contain (critical) errors, the amount of research potentially building on false grounds remains worrisome.

A spreadsheet with all results can be found in the Supplementary Material (S2 File). This table lists all cell lines in the ICLAC database and the number of articles in the primary and secondary literature reporting on these cell lines, both for search method 1 and 2. In addition, the mean citation rate for articles in the primary literature is given as well as information on the temporal distribution of the secondary literature (the first and last year in which articles are published as well as the year in which most of the secondary literature on this cell line appeared). Given the fact that citation distributions tend to form (truncated) bell-shaped curves, this information provides reasonable insight in the temporal distribution of the secondary literature. The data is listed per cell line and not summarised, as this approach could lead to double counts.

The total number of research articles on cells can be estimated between 4.5 and 5 million (see methods). Therefore, the contaminated primary literature makes up a little under 0.8% of the total literature on cells, while the (potentially) contaminated secondary literature can be estimated in the order of 10% of the total research output in this area. However, we should stress that our aim is to measure the size of the problem. The sample undoubtedly contains false positives and is hence not suitable to identify individual contaminations.

### Closer inspection of primary literature

An objection to our findings might be that our general search methods do not provide a proper overview of how specific misidentified cell lines actually affect research. To get a deeper understanding of how knowledge based on misidentified cell lines spreads through the literature, we present three case studies in which we tracked the publications concerning a single cell line or a family of cell lines. All three are misidentified cell lines for which no original stock was reported and were selected at random from the ICLAC database.

#### ALVA-31

This cell line was originally established in 1993 as a human prostate carcinoma [35], but was found to be identical to a different human prostate carcinoma, the PC-3 cell line, in 2001 [36, 37]. We found 56 articles referring to ALVA-31, which are in turn cited by 2615 articles. Of these 56 primary articles, 22 were published after the misidentification of the ALVA-31 cell line was discovered. On closer inspection of those 22 articles, it appears that the ALVA-31 cell line was actually used in 20 of them, while only two articles mention the cell line’s misidentification. Remarkably, the most recent articles describing research based on ALVA-31 cells are published in 2016, fifteen years after the misidentification was reported.

In this case, one could argue that it might do little harm to use ALVA-31 cells, while actually working with PC-3 cells, because both are human prostate carcinoma and share many characteristics. However, in some cases, even researchers themselves argue that the precise identity of ALVA-31 is essential: “To exclude a cell type-specific effect, we extended ALVA-31 studies to other human PCa cell types” [38]. Subsequently, the authors explain how they used PC-3 cells in additional studies to ‘exclude cell type-specific effects’; in effect comparing two identical cell lines.

#### Thymic cell lines

In a 1994 report, the establishment of a group of novel thymic cell lines (F2-4E5, F2-5B6, P1-1A3 and P1-4D6) [39] was announced. In a report by MacLeod et al. [40], the cell lines were found to be misidentified, having been derived in fact from a liver carcinoma. In total, 69 articles were found that refer to these cell lines, in turn cited by 2092 articles. Of the primary articles, 43 were published after the report by MacLeod et al. and the most recent one was published only in late 2016 [41]. Of the fifteen most recent articles referring to the 1994 report, thirteen actually refer to it because they use the cell lines, all thirteen reporting research on thymic cells, without mentioning any knowledge of the misidentification of these cell lines. The other two articles refer to the establishing article for the sake of the method used in it to establish novel cell lines.

#### JCA-1

The JCA-1 cell line was originally established in 1990 [42] and found to be misidentified in 2001 by van Bokhoven et al. [43], who showed that the cells in fact are derived from a bladder carcinoma rather than a prostate carcinoma. We found 64 articles referring to the establishing paper or explicitly mentioning JCA-1 in their title, key words or abstract. In turn, these articles are cited by 3352 articles. Of the primary articles, 18 appeared after the report by van Bokhoven et al. In contrast to the cell lines discussed previously, there seems to be no contemporary usage of JCA-1 in scientific research: the most recent article describing research using this cell line dates from 2009. However, also in this case, several articles were published reporting to use ‘prostate cancer cell lines’, after it became known that JCA-1 actually originated from bladder carcinoma. In fact, as we verified in the full text, of the 18 articles published after the report by van Bokhoven et al. [43], only 3 show awareness of the fact that the line had been misidentified. In contrast, 14 simply stated to have used the JCA-1 cell line, the vast majority explicitly referring to them as prostate cancer cells.

As these case studies show, merely listing a cell line as misidentified does not deter scientists from using it. This constitutes additional evidence for the claim that avoiding future contaminations does not form a complete solution to the issue of cell line misidentification. Instead, demonstrably misidentified cell lines continue to have an impact on research, either directly because scientists keep using them, or indirectly because scientists build on previous research employing misidentified lines. (Additional case studies on the consequences of using misidentified cell lines can be found at the ICLAC webpage [19].)

### A transitory problem?

One might wonder whether the contamination of the research literature is mainly a problem of the past, given that the first concerns about misidentified cell lines were expressed half a century ago [9, 10] and that numerous initiatives have tried to alleviate the problem since.

**Fig 2:**
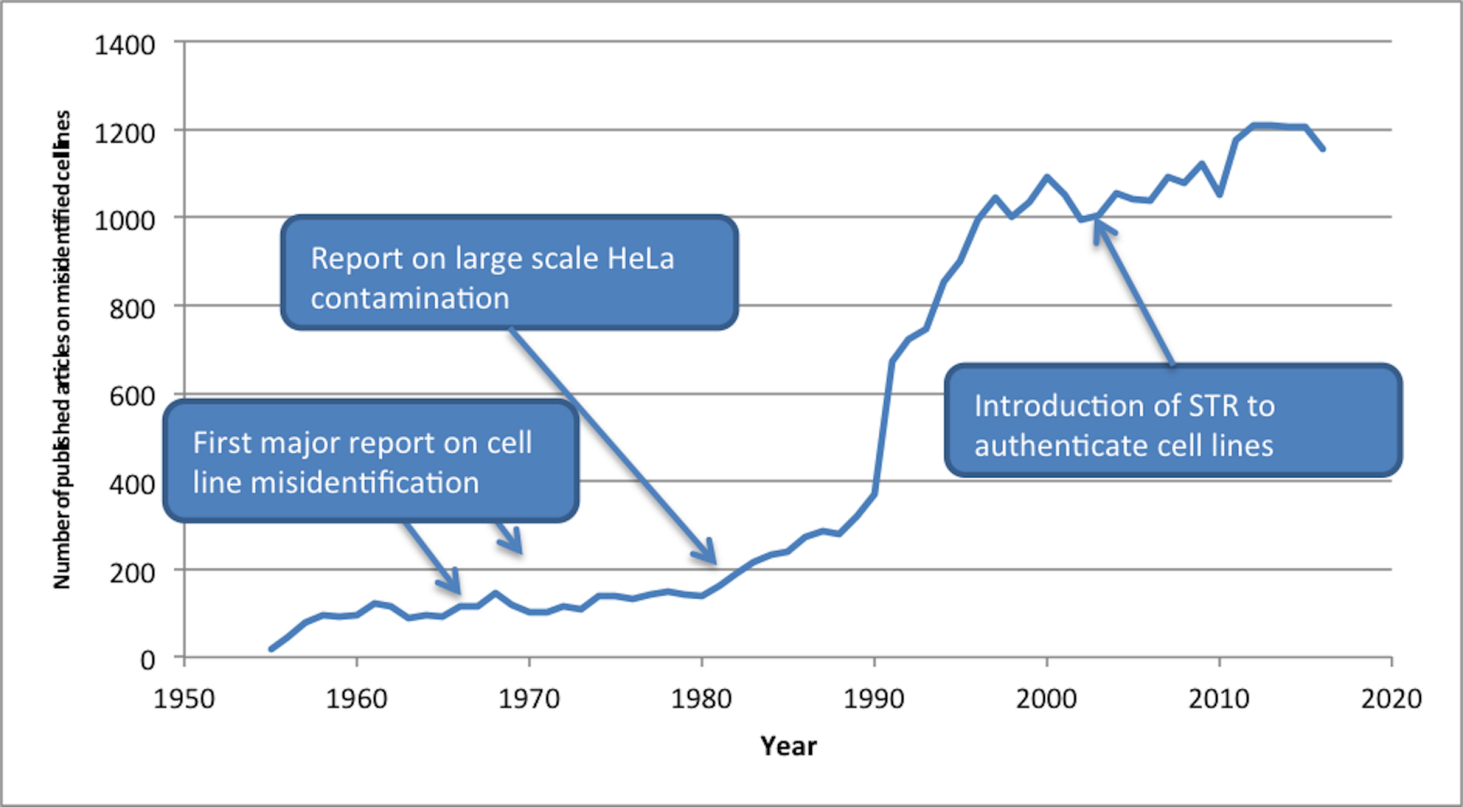
The distribution of the contaminated primary literature over the years. The graph includes references to the first report on intraspecies cell line misidentification [10], a major list of misidentified cell lines based on HeLa contaminations [44] and the introduction of Short Tandem Repeat (STR) as technique for cell line authentication [45].

Based on the set of 32,755 records of primary contaminated literature, we analysed the publication dates of the articles. The majority of the articles, 57%, were written since 2000 and the number of articles using misidentified cell lines is still growing (see Fig 2). Clearly, the problem is definitely not one of the past, but is very relevant to contemporary science, with 58 new articles based on contaminated literature appearing even as recently as February 2017.

Fig 2 indicates three moments in history when cell line contamination became evident. First, through the work of Stanley Gartler it became possible to detect intraspecies cell contamination, after which several of such contaminations involving HeLa cells were reported in *Nature* in 1968 [9, 10]. Second, cell culture contamination was put on the global research agenda by the work of Walter Nelson-Rees et al. in the 1970s [7, 8], culminating in a list of contaminated cell cultures in *Science* in 1981 that demonstrated large-scale contamination of cell cultures by HeLa cells [44]. From this point on, it could be expected that most scientists working in those areas of research frequently employing cell cultures, were aware of the potential issues with their research material. However, the vast majority of research papers based on misidentified cell lines was published after this point in time. Even after the introduction of STR in 2001 [45], the annual number does not decrease.

Similar to the primary literature, the number of articles in the secondary literature is also still growing. In 2016, over 40,000 papers were published that referred to primary contaminated literature. In addition, from the information in the Supplementary Material (S2 File), we conclude that the majority of misidentified cell lines continue to contaminate the secondary literature in 2017 (251 cell lines for search method 1 and 232 cell lines for search method 2), while dozens of cell lines created most of their secondary literature in the past two years (38 for search method 1 and 87 for search method 2). Moreover, we conclude that many cell lines (108 for search method 1, 87 for search method 2) have generated contamination in secondary literature for a period of more than 25 years, with articles appearing long after it became known that the cell line was misidentified. Hence the contamination of the literature through reference to articles using misidentified cell lines remains a very topical problem.

### A peripheral problem?

Another objection to our findings could be that cross-contamination occurs particularly in regions with new or emerging research communities, in which levels of training or access to testing facilities may be limited. For example, several recent publications indicate levels of cell line contamination for China between 25% [13] and 46% [46] and demonstrate that of all ‘new’ cell lines developed in China 85% actually turned out to be HeLa cells [13].

**Fig 3:**
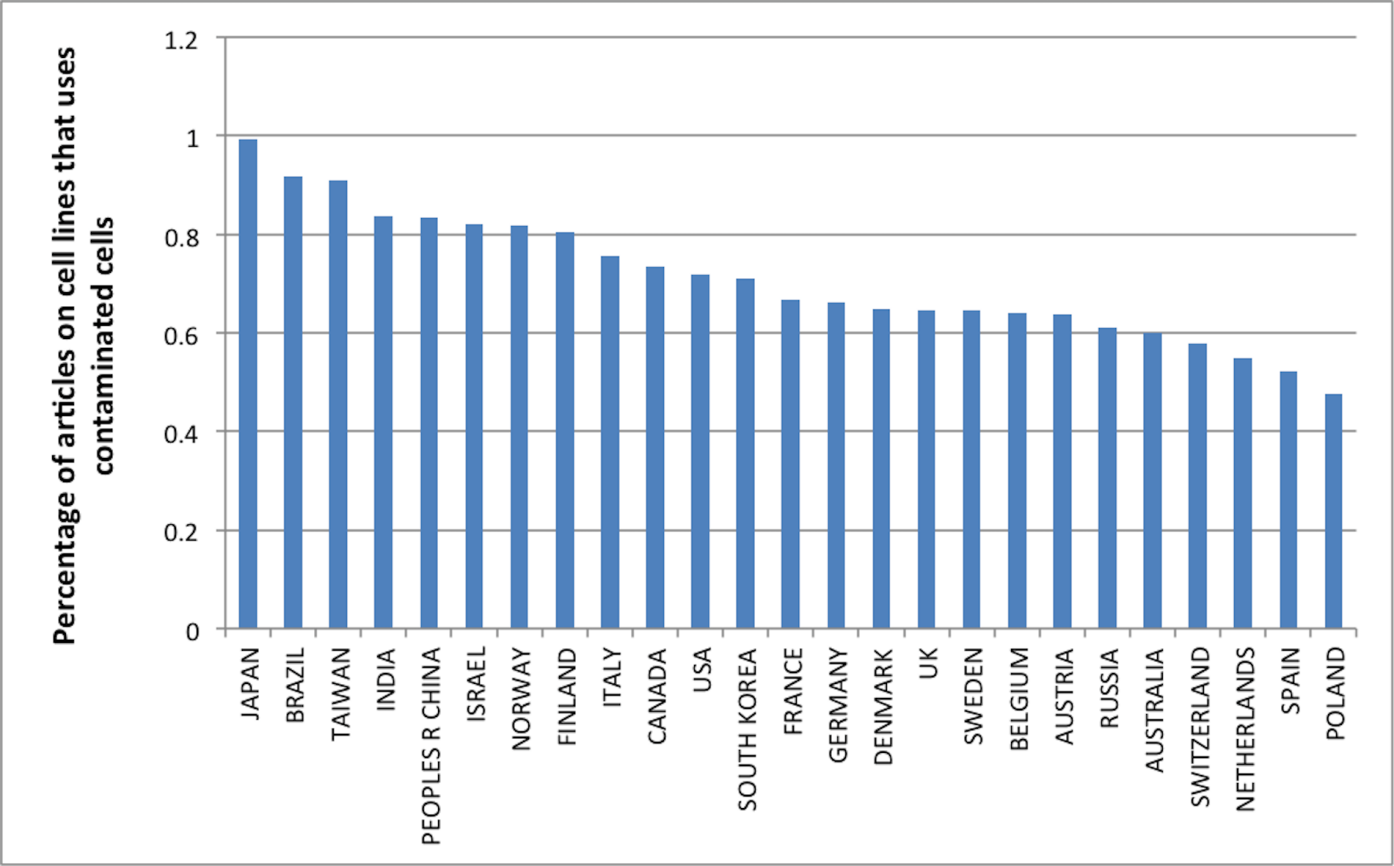
The percentage of contaminated primary articles as a fraction of the total number of articles on cells per country. The figure includes the 25 countries with the largest absolute number of articles in the contaminated primary literature.

However, the majority of the articles using misidentified cell lines originate from countries holding well-established research traditions (e.g. US, Japan, Germany). Relative to their share of total research output, authors from these countries often perform research on misidentified cell lines. In fact, mainly due to their enormous share of total literature on cell lines, over 36% of all contaminated primary literature stems from the US. Fig 3 shows the percentage of contaminated primary articles as a fraction of the total number of articles on cells per country (see Supplementary Materials S2 File for data). It includes the 25 countries with the largest share of the contaminated primary literature. In this list, we see countries holding excellent research reputations ranking high. Hence, the problem does not only occur in regions with low standards of quality and diligence in research, but is also a problem in countries that hold excellent research reputations. Nevertheless, an analysis of the literature for the past five years showed a dramatic rise of China’s share in the contaminated literature, confirming recent worries expressed in the literature [13].

**Fig 4:**
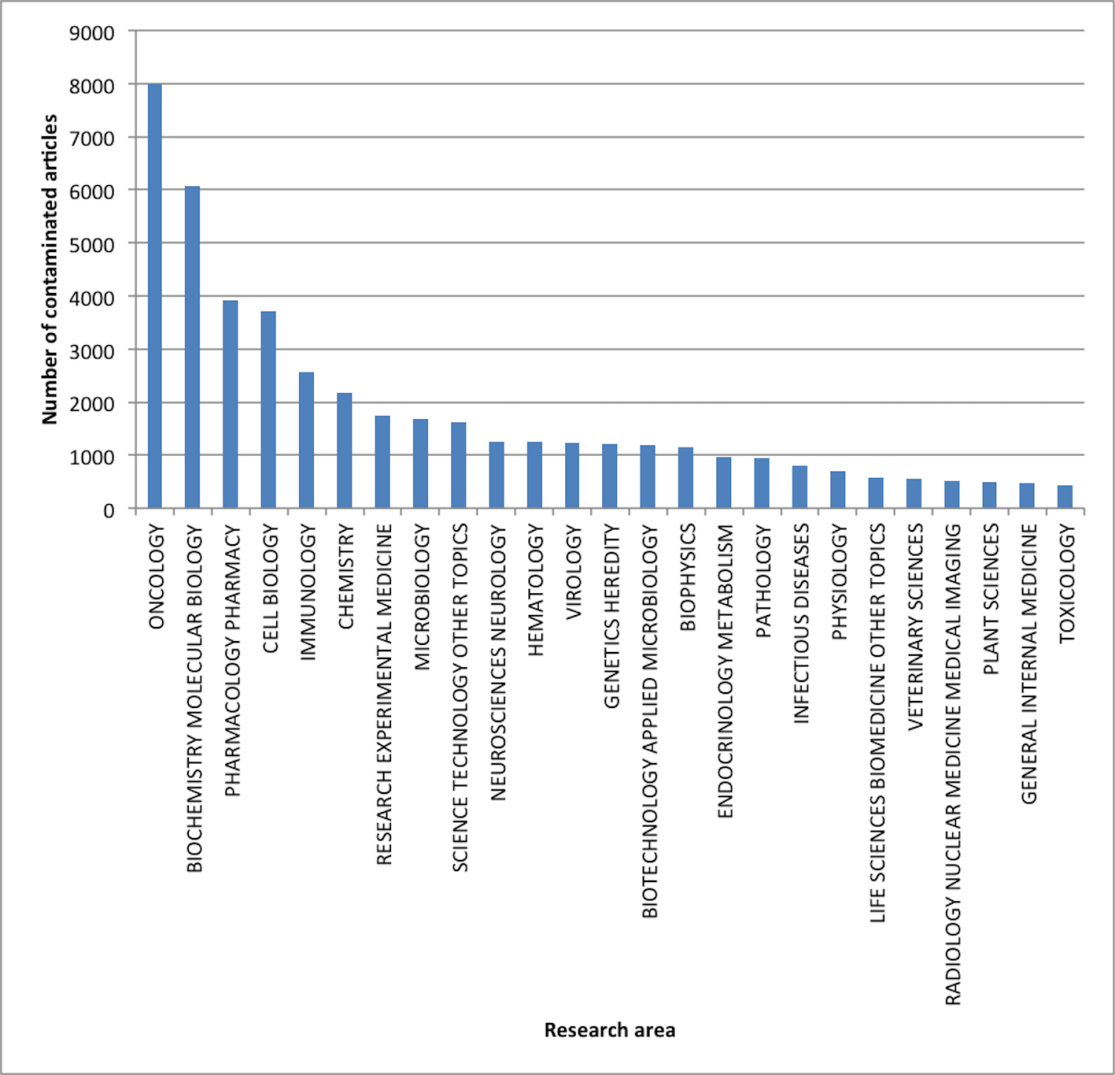
The distribution of contaminated primary literature over the research areas as defined by *Web of Science*. Only the 25 most affected research areas are included.

Last, we analysed which research disciplines were most affected by the use of misidentified cell lines. Fig 4 shows the distribution of contaminated articles over the various research areas as defined by WoS. Among the contaminated primary literature, oncology, biochemistry/molecular biology, pharmacology and cell biology are most affected, confirming concerns about medical applications.

However, analysis of citations obtained by the primary literature indicates that the secondary literature spreads to a much more diverse range of research areas. The articles in the secondary literature originate also in fields rarely using cell lines for their research, such as psychiatry, engineering and agriculture science, see Fig 4. Consequently, the impact of misidentified cell cultures may spread to non-biomedical fields and affect scientists that are not as trained to judge the validity of research on misidentified cell lines.

## Discussion

### Potential counter arguments

Our results seem to present worrying problems for the biomedical sciences. Although the issue of misidentified cell lines has long been known, its effect on the scientific literature has not been properly recognised, let alone properly treated [47, 48]. However, various arguments have been presented to suggest that papers based on misidentified cell lines are still valuable and that no remedial action is needed.

First, it has been asserted that, in some cases, the origin or specific characteristics of a cell line might be of little influence on the results of an experiment. Indeed, in some cases all that a researcher needs is ‘a cell line’, independent of type, origin or status. In fact, this argument was already mentioned by Gartler in 1968 to put his findings into perspective [10]. To be sure, we acknowledge that not all 32,755 articles that we found contain critical errors. However, this is not a valid argument *not* to label articles that employ misidentified cell lines, for two reasons. To begin with, it is currently up to every individual scientist to judge the status of an article every time they cite or read it, by first checking the ICLAC database of misidentified cell lines to see whether any of the cell lines used in an article are in this database, and subsequently judging the influence that the misidentification may have on the results. This is a cumbersome and unlikely assumption about how researchers cite their literature, given the low levels of awareness indicated in our analysis. In addition, the secondary literature spreads into research fields that do not commonly use cell lines for their research. It is particularly doubtful whether scientists from these fields are aware of the potential issues with research on cell lines and whether they are in the position to make informed decisions about the validity of the claims in research articles based on misidentified cell lines.

Second, it has been argued that no remedial action is needed, as the problem will be addressed by new verification techniques. Similarly, it has been argued that the problem is already widely known, that scientists may be expected to implement effective lab protocols and be sufficiently critical about cell lines and their literature. Hence contaminated literature should have faded beyond the time horizon of literature considered relevant for current research and have disappeared from the relevant research record [49]. However, there is no sign of any ‘fading away’ of the problem. As we demonstrated, both the number of articles using misidentified cell lines, and the number of articles referring to them are still growing. Moreover, as demonstrated in the case studies, scientists show little awareness of the fact that cell lines may be misidentified. The citation analysis of the primary literature shows that articles keep being cited long after misidentifications have been reported, with over 40,000 articles citing contaminated research articles in 2016, including hundreds of citations to primary contaminated literature published decades ago.

### Practical measures

Over the past decades thorough attention has been paid to the improvement of authentication testing for cell lines. Authentication of cell lines, and hence the ability to demonstrate cross contamination, became possible by the introduction of genetic markers by Gartler in 1967 [9]. Subsequently, many techniques for cell line authentication were introduced, starting off with inspection of banded marker chromosomes [8], and the visualization of chromosomal pattern and architecture in general [50], subsequently followed by the methods of Human Leukocyte Antigen (HLA) typing [51], enzyme polymorphisms [52] and DNA polymorphisms [53]. More recently, the techniques of ‘DNA fingerprinting’ [54] and the usage of locus-specific probes were introduced. Finally, this led to the now accepted standard method of short tandem repeat profiling [45]. As has been pointed out recently, the techniques for proper cell line authentication are now widely available [55]. However, implementation of these techniques is still falling short for multiple reasons, including time and financial constraints, lack of training and lack of (international) standards [55].

Despite measures to authenticate new and existing cell lines [27], research based on the wrong cells is still present in the literature and in fact continues to be published. Some form of precautionary labelling of contaminated articles seems unavoidable. However, this remedial action should be proportionate and not cause unnecessary damage. For some individual scientists, research departments, or scientific journals, rash measures could turn out to be painful. Indeed, some researchers have authored over a hundred articles in our set of contaminated primary literature. Even though the problem with these articles almost exclusively [56] falls under the heading of ‘*honest error’*, with no intention to deceive, notifying all those articles as potentially erroneous, or worse: retracting them, would have a disproportionate impact on several scientists’ careers. This would undermine, rather than support, an effective clean-up operation. However, in addition to catching cell line contamination at the source, initiatives to label contaminations ‘downstream’ in the published literature are direly needed. We can make several suggestions.

First, notifications should be posted alongside previously published articles using misidentified cell lines. This could be done in the form of ‘expressions of concern’, which are described as “*Neither retractions nor corrections*, *they alert readers that there may be an issue with a paper*, *when the full story is not yet clear*.*”* [57] If clear and uncontended, the consequences of the misidentification for the article’s conclusions could be reported, but otherwise the expression of concern could merely state: “Cell line X in this study is known to be misidentified and is actually Y. See Z for more information.” The interpretation of this warning is then entirely up to the expert reader. Such notifications would also serve to preserve as much valuable data as possible: data reported on a misidentified cell line might still be entirely valid, provided the real origin of the cell line is clear. Hence it might be a waste of funds and efforts to automatically dismiss these data. In cases where the use of these cell lines leads to (severely) false conclusions that could have a major impact on future research, articles could be retracted. For recent cases, a system of self-retractions, as proposed by Fanelli [58], could be employed.

Second, to allow for simple future identification of articles using misidentified cell lines, we recommend that authors mention the employed cell lines in easily searchable parts of their article, such as the keywords or abstract. Some journals have already suggested measures in this direction, but implementation seems to be slow [30]. However, some journals have currently installed a system of Research Resource Identifiers (RRIDs), which might assist in tackling the cell line misidentification issues [59]. Alternatively, a system of cross-reference between databases of cell lines and scientific journal publications could be set up. Linking the NCBI databases of ‘BioSamples’ and ‘research articles’ would be a natural candidate for such a system. In similar ways, the Cellosaurus database, the ICLAC database and Research Resource Initiative are already cross-linked.

In addition, better use could be made of paper trails for cell line provenance [60]. A clear and complete overview of the origin of a cell line, as well as the various verification tests, the experiments that it has been part of, and the results that these yielded, would be of great benefit in examining the status and quality of a cell line. In addition, this would allow for easy identification of potentially erroneous research when a cell line is found to be misidentified.

Besides being of use in terms of recognition of erroneous research, the paper trail might also serve other purposes, such as mapping the existing knowledge on a certain cell line (thereby also allowing for simple identification of knowledge gaps) and providing a stage for the publication of negative results of experiments on cell lines. The publication of such results has long been proposed as a way of fostering integrity in research [61].

Nearly half a century after the first concerns about misidentified cell lines, the initiatives to improve authentication need to be complemented by attention to the already contaminated literature. Our analysis shows that the task is sizeable and urgent.

## Supporting information

S1 File Supporting information. Names of cell lines that were adjusted for the search in method 2 or that were excluded from the search and included research areas (as defined by WoS) in search method 2.
(DOCX)

S2 File Supplementary material. Data by country and by cell line.
(XLSX)
