# Supplementary material for: The ghosts of HeLa: How cell line misidentification contaminates the scientific literature
Source: PLoS One. 2017 Oct 12;12(10):e0186281. doi: 10.1371/journal.pone.0186281 (PMC5638414; doi:10.1371/journal.pone.0186281)
Supplement: S1 File — Names of cell lines that were adjusted for the search in method 2 or that were excluded from the search and included research areas (as defined by WoS) in search method 2. (DOCX) [file pone.0186281.s001.docx]

Supporting information

| **Cell lines that were searched via: "XX cell*" (instead of just the cell name)** | **Cell lines that were excluded from the search**  because of unavoidable confusion. |
| --- | --- |
| AO cell* | 2008/C13*5.25 (C13, OV2008/C13) |
| C13 cell* | 222 |
| CaVe cell* | 2474/90 |
| CCL3 cell* | 41M |
| CO cell* | DAPT |
| DD cell* | HBC |
| EH cell* | LU |
| EPC cell* | MT-1 [Multiple cell lines with same name, see Cellosaurus] |
| EU-1 cell* | MT-3 [Multiple cell lines with same name, see Cellosaurus] |
| EU-7 cell* | OF |
| FL cell* | REPC |
| FQ cell* | T1 |
| HAG cell* | T-1 |
| Hut cell* | T406 [Multiple cell lines with same name, see Cellosaurus] |
| K1 cell* | YAA |
| K2 cell* |  |
| KB cell* |  |
| MDS cell* |  |
| OE cell* |  |
| OST cell* |  |
| RB cell* |  |
| T-9 cell* |  |
| TE-2 cell* |  |
| TE-3 cell* |  |
| WISH cell* |  |
| YAP cell* |  |
| YJ cell* |  |

Table S1: names of cell lines that were adjusted for the search in method 2 or that were excluded from the search.

**Included research areas (as defined by WoS) in search method 2:**

| ONCOLOGY |
| --- |
| BIOCHEMISTRY MOLECULAR BIOLOGY |
| CELL BIOLOGY |
| IMMUNOLOGY |
| HEMATOLOGY |
| PHARMACOLOGY PHARMACY |
| RESEARCH EXPERIMENTAL MEDICINE |
| SCIENCE TECHNOLOGY OTHER TOPICS |
| GENETICS HEREDITY |
| PATHOLOGY |
| VIROLOGY |
| NEUROSCIENCES NEUROLOGY |
| BIOPHYSICS |
| BIOTECHNOLOGY APPLIED MICROBIOLOGY |
| ENDOCRINOLOGY METABOLISM |
| CHEMISTRY |
| VETERINARY SCIENCES |
| FISHERIES |
| MICROBIOLOGY |
| PHYSIOLOGY |
| GENERAL INTERNAL MEDICINE |
| SURGERY |
| LIFE SCIENCES BIOMEDICINE OTHER TOPICS |
| UROLOGY NEPHROLOGY |
| RADIOLOGY NUCLEAR MEDICINE MEDICAL IMAGING |

The restriction of the search to these research areas resulted in the exclusion of 2966 articles from our analysis.
